# Economic evaluation of tislelizumab versus chemotherapy as second-line treatment for advanced or metastatic esophageal squamous cell carcinoma in China

**DOI:** 10.3389/fphar.2022.961347

**Published:** 2022-11-16

**Authors:** Fenghao Shi, Zixuan He, Hang Su, Lin Wang, Sheng Han

**Affiliations:** ^1^ International Research Center for Medicinal Administration, Peking University, Beijing, China; ^2^ School of Pharmaceutical Sciences, Peking University, Beijing, China; ^3^ School of International Pharmaceutical Business, China Pharmaceutical University, Nanjing, China

**Keywords:** cost-effectiveness analysis, esophageal squamous cell carcinoma, tislelizumab, second-line treatment, China

## Abstract

**Background and purpose:** The latest RATIONALE-302 trial (NCT03430843) showed that tislelizumab therapy significantly improved overall survival benefits for patients with advanced or metastatic esophageal squamous cell carcinoma (ESCC) compared with traditional chemotherapy. This study aimed to compare the cost-effectiveness of tislelizumab versus chemotherapy as a second-line treatment for advanced or metastatic ESCC in China.

**Methods:** A partitioned survival model was developed to predict patients’ lifetime quality-adjusted life-years (QALYs) and incremental cost-effectiveness ratio (ICER) from the Chinese healthcare payers’ perspective. We extracted efficacy and safety data from the RATIONALE-302 trial and the local cost and resource use data from online databases and published studies. One-way sensitivity analysis (OWSA) and probabilistic sensitivity analysis (PSA) were performed to explore model uncertainty.

**Results:** Compared with chemotherapy, tislelizumab generated a higher cost (US$ 10211.78 vs. US$ 7294.72) but yielded more QALY (0.78 vs. 0.51 QALYs). The ICER for tislelizumab was US$11073.85 per QALY gained. The PSA results indicated that the probability of tislelizumab being economical was 76% under a willingness-to-pay (WTP) threshold of 1.5 times per capita GDP ($17915) in China.

**Conclusion:** Tislelizumab could be a promising cost-effective strategy as the second-line treatment for patients with ESCC compared with chemotherapy in the Chinese setting.

## Introduction

Esophageal cancer (EC) is one of the commonest malignant tumors associated with distinct morbidity and mortality globally (Sung et al., 2021). The two main histological types of oesophageal cancer include esophageal squamous cell carcinoma (ESCC) and oesophageal adenocarcinoma (Abnet et al., 2018). Studies indicated that the incidence of EC in Asia and Southern Africa outclassed other regions, with approximately 90% of esophageal cancer patients diagnosed in Asia and 30% in the US and other Western countries. China bears a rather heavy disease burden in the high-risk areas, with more than 3,20,000 esophageal cancer cases occurring in 2020 (Lagergren et al., 2017). (Zhang et al., 2012).

Since the clinical symptoms of early ESCC are not distinctive, most patients are at an advanced stage when diagnosed with ESCC. Platinum drugs combined with 5- fluorouracil or paclitaxel are recommended as the standard first-line treatment option for advanced or metastatic ESCC. A retrospective study showed that paclitaxel plus cisplatin (TP) results in similar median progression-free survival (PFS) compared with 5-fluorouracil plus cisplatin (FP) (7.9 months versus 6.5 months) in patients with advanced ESCC. However, such chemotherapeutic regimens’ clinical benefits are limited, with a median overall survival of less than 1 year (Liu et al., 2016).

In recent years, Immune checkpoint inhibitors (ICIs) that target programmed cell death protein 1(PD-1) or programmed cell death ligand 1 (PD-L1) have shown outstanding performance in esophageal cancer therapy, which enhances the anti-tumor activity across esophageal cancer. Existing randomized studies that evaluate PD-(L)1 blockade in the second-line treatment of ESCC patients demonstrate a significant OS improvement in anti-PD-(L)1 compared with chemotherapy (Kato et al., 2019; Shah et al., 2019; Huang et al., 2020; Kojima et al., 2020; Luo et al., 2021).

Tislelizumab, a fully humanized, immunoglobulin G4 (IgG4) monoclonal antibody specific for human PD-1, is designed to limit antibody-dependent phagocytosis and to minimize binding to FcgR on macrophages (Xu et al., 2020). The RATIONALE-302 trial (NCT03430843), an open-label phase III clinical study covering 512 patients across 11 countries/regions, showed that treating ESCC patients with tislelizumab was associated with longer overall survival (8.6 v 6.3 months), higher objective response rate (20.3% v 9.8%) and a more durable anti-tumor response (7.1 months v 4.0 months). This trial demonstrated a clinically meaningful efficacy and a manageable safety profile of tislelizumab compared with traditional chemotherapy (Shen et al., 2022). Although tislelizumab showed superior clinical benefit, its cost-effectiveness in treating ESCC awaits further investigation. To this end, this paper evaluates the cost-effectiveness of tislelizumab vs. traditional chemotherapy regimens as the second-line treatment for ESCC from the Chinese healthcare payers’ perspective, aiming to inform policy and clinical practice.

## Materials and methods

### Model structure

**FIGURE 1:**
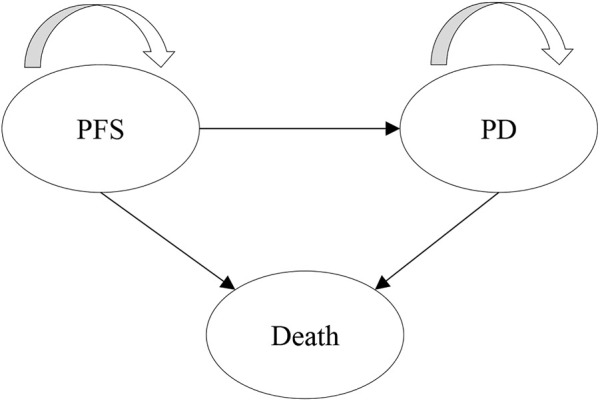
Model structure.

A partitioned survival model (PartSA) was developed to evaluate the clinical cost-effectiveness of tislelizumab compared with chemotherapy in China. As suggested and recognized by the comparative studies, our PartSA model has included three states: progression-free survival (PFS), progressive disease (PD), and death (Figure 1) (Minacori et al., 2015). To evaluate the lifetime impact of treatment on the patients, we simulated until 99% of patients in the tislelizumab group and the chemotherapy group were dead. We used standard survival parametric functions, log-logistic and log-normal, to fit the OS and PFS survival curves. Outputs of the model contain long-term cost, quality-adjusted life-years (QALYs), and incremental cost-effectiveness ratio (ICER). As suggested by the Guidelines for pharmacoeconomic evaluation in China and comparative studies, we applied a willingness-to-pay (WTP) threshold of 1.5 times China’s national per capita GDP (US$17915 per QALY in 2021) to test the cost-effectiveness of second-line tislelizumab (China Market Press, 2020) (Cai D.et al., 2021). The study set a 10-year time horizon with each simulation cycle of 3 weeks, aligning the design of the RATIONALE-302 trial. All analyses were conducted using Microsoft Excel 2021.

### Patients and treatment

The population for the economic evaluation is aligned with the patient population targeted by the RATIONALE-302 trial: adult (age ≥ 18 years) ESCC patients with progressed after the first-line treatment. Detailed information on the clinical trials can be found in Supplementary Table S1.

Patients with ESCC were randomized into two groups: 1) tislelizumab group: tislelizumab 200 mg once every 3 weeks; 2) single-agent chemotherapies group: 125 mg/m^2^ irinotecan once every 2 weeks, 135–175 mg/m^2^ paclitaxel once every 3 weeks or 75 mg/m^2^ docetaxel once every 3 weeks.

### Clinical data inputs

The clinical efficacy and safety data of tislelizumab and chemotherapies were extracted from the RATIONALE-302 study (Shen et al., 2022). WebPlotDigitizer (https://automeris.io/WebPlotDigitizer/) was used to pick points from the OS and PFS curves to obtain the OS rate and PFS rate. The pseudo-individual patient data (IPD) was reconstructed with the recommendation of Guyot and fitted using the standard parametric models in IPDfromKM (Guyot et al., 2012; Liu et al., 2021). We explored Log-normal, Gamma, Weibull, Gompertz, Exponential, Log-logistic, and Generalized gamma functions to fit the curve (Latimer, 2011). The best fitting distribution was selected by the lowest value of the Bayesian information criterion (BIC), the Akaike information criterion (AIC) and visual inspection (Supplementary Table S2; Supplementary Figures S1–S4). As a result, the log-logistic function was used to simulate the OS curves of the two schemes, the log-normal function was used to simulate the PFS curves of tislelizumab, and the generalized gamma function was used to simulate the PFS curves of chemotherapy. All the survival curve simulation results were shown in Table 1.

**TABLE 1:** Survival parameters.

KM	Best fitting	Survival parameters
OS for tislelizumab	Log-logistic	Shape = 1.52614, Scale = 8.63638
PFS for tislelizumab	Log-normal	Meanlog = 1.02782, sdlog = 1.04613
OS for chemotherapy	Log-logistic	Shape = 1.85767, Scale = 6.23685
PFS for chemotherapy	Generalized gamma	Mu = 0.606652, Sigma = 0.811023, Q = −0.603264

### Cost and utility inputs

The study applied the Chinese healthcare system’s perspective that only accounts for direct medical care costs, including drug acquisition and administration of tislelizumab and chemotherapy, follow-up costs, best supportive care (BSC), terminal care in end-of-life and management of treatment-related grade ≥3 serious adverse events (SAEs). The drug unit costs were the mean bidding price derived from the China Drug Bidding Database on YAOZH (yaozh.com) and converted into 2021 USD. Treatment regimens, the proportion of patients using each scheme, and the incidence of severe adverse events were extracted from the RATIONALE-302 trial (Shen et al., 2022). The costs of follow-up, best supportive care, and end-of-life care were derived from published sources (Cai H.et al., 2021). Please refer to Table 2 for details.

**TABLE 2:** Model input parameters.

Model input	Base case	Range	Distribution	Reference
Drug costs				
Tislelizumab (100 mg)	427.73	320.80–427.73	Gamma	YAOZH
Docetaxel (20 mg)	21.60	7.98–144.54	Gamma	YAOZH
Irinotecan (100 mg)	119.39	87.76–284.56	Gamma	YAOZH
Paclitaxel (30 mg)	10.86	5.60–72.17	Gamma	YAOZH
Investigator’s choice of chemotherapies				
Docetaxel cases (%)	53 (20.70%)	—	Dirichlet	Shen et al. (2022)
Irinotecan cases (%)	118 (46.10%)	—	Dirichlet	Shen et al. (2022)
Paclitaxel cases (%)	85 (33.20%)	—	Dirichlet	Shen et al. (2022)
Body surface area (BSA, m^2^)	1.72	1.50–1.90	Gamma	Zhang et al. (2021)
Follow-up cost	7.46	6.52–8.47	Gamma	Cai D.et al. (2021)
Best supportive care cost	167.29	133.83–200.75	Gamma	Cai H.et al. (2021)
End-of-life care cost	1460.30	1168.24–1752.36	Gamma	Cai D.et al. (2021)
Utility				
PFS	0.741	0.593–0.889	Beta	Zhang et al. (2020)
PD	0.581	0.465–0.697	Beta	Zhang et al. (2020)
Discount	5%	0%–8%	Constant	

As the quality of life information was not collected in the RATIONALE-302 study, we applied utility values of PFS and PD status based on the relevant literature, as well as costs and disutilities resulting from AEs (Zhang et al., 2020). Please refer to Supplementary Table S3.

### Sensitivity analyses

We conducted both deterministic sensitivity analysis (DSA) and probabilistic sensitivity analysis (PSA) to check the robustness. The DSA undertook a series of one-way sensitivity analyses shown as Tornado diagrams that graph the variable sequentially with the most considerable impact on the economic results. In the PSA, we assumed cost parameters obeyed the gamma distribution, and the incidence of AEs and utility parameters followed the beta distribution. In addition, all the survival parameters were assessed through Cholesky decomposition. 5000 Monte Carlo iterations were performed to assess the overall model uncertainty. Accordingly, the incremental ICER scatterplot and a cost-effectiveness acceptability curve (CEAC) representing uncertainty were derived.

## Results

### Base-case analysis

Table 3 shows the base case results of tislelizumab compared with chemotherapy. The lifetime cost of tislelizumab was higher than that of chemotherapy ($10211.78 vs. $7294.72). The health outcomes of tislelizumab and chemotherapy were 0.78 QALYs and 0.51 QALYs, respectively. Compared with the chemotherapy regimen, tislelizumab yielded an additional 0.26 QALYs at an incremental cost of $2917.06, resulting in an ICER of $11073.85 per QALY. Under a WTP threshold of 1.5 times China’s 2021 GDP per capita ($17915), tislelizumab was a cost-effective strategy.

**TABLE 3:** Base case results from the cost-effectiveness analysis.

Treatment	Cost	QALY	Incremental cost	Incremental QALY	ICER
Chemotherapy	7294.72	0.51			
Tislelizumab	10211.78	0.78	2917.06	0.26	11073.85

### One-way sensitivity analysis

**FIGURE 2:**
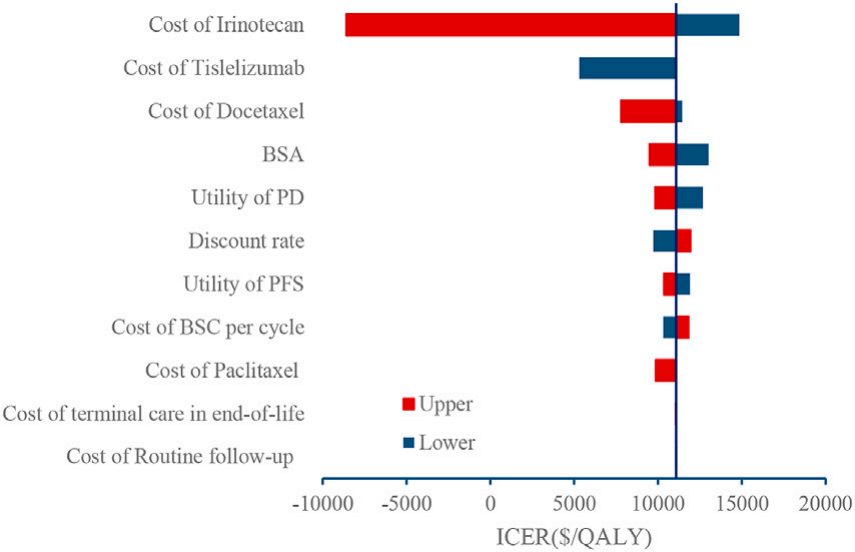
Tornado diagram.

The results of deterministic sensitivity analyses are presented in Figure 2. The cost of irinotecan and tislelizumab are the main factors of ICER. Tislelizumab could become a dominant treatment strategy when the cost of irinotecan equals $284.56. Even if the cost of irinotecan dropped to $284.56, the ICER of tislelizumab was $14841.52 per QALY, which remains cost-effective under the threshold.

### Probabilistic sensitivity analysis

**FIGURE 3:**
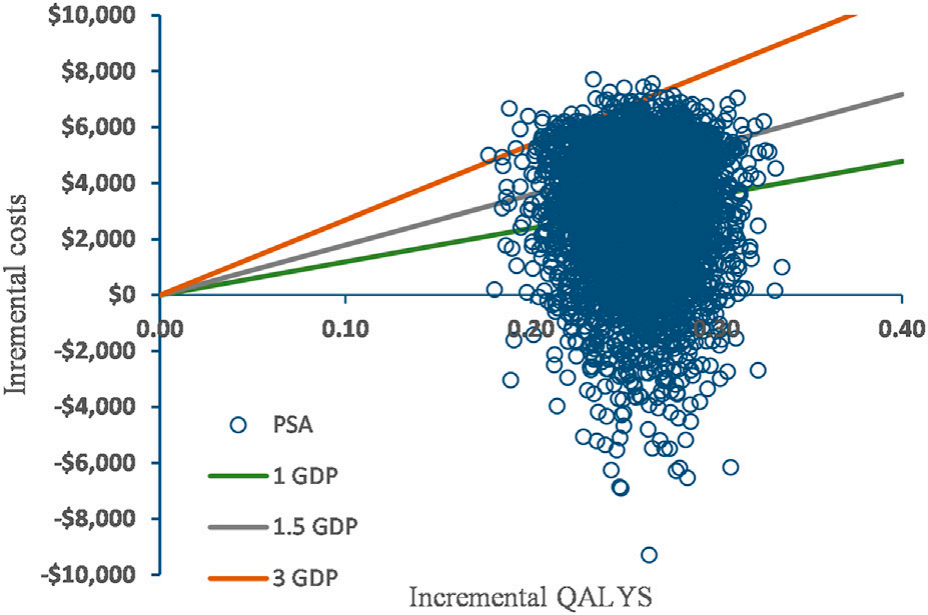
Scatter plot.

**FIGURE 4:**
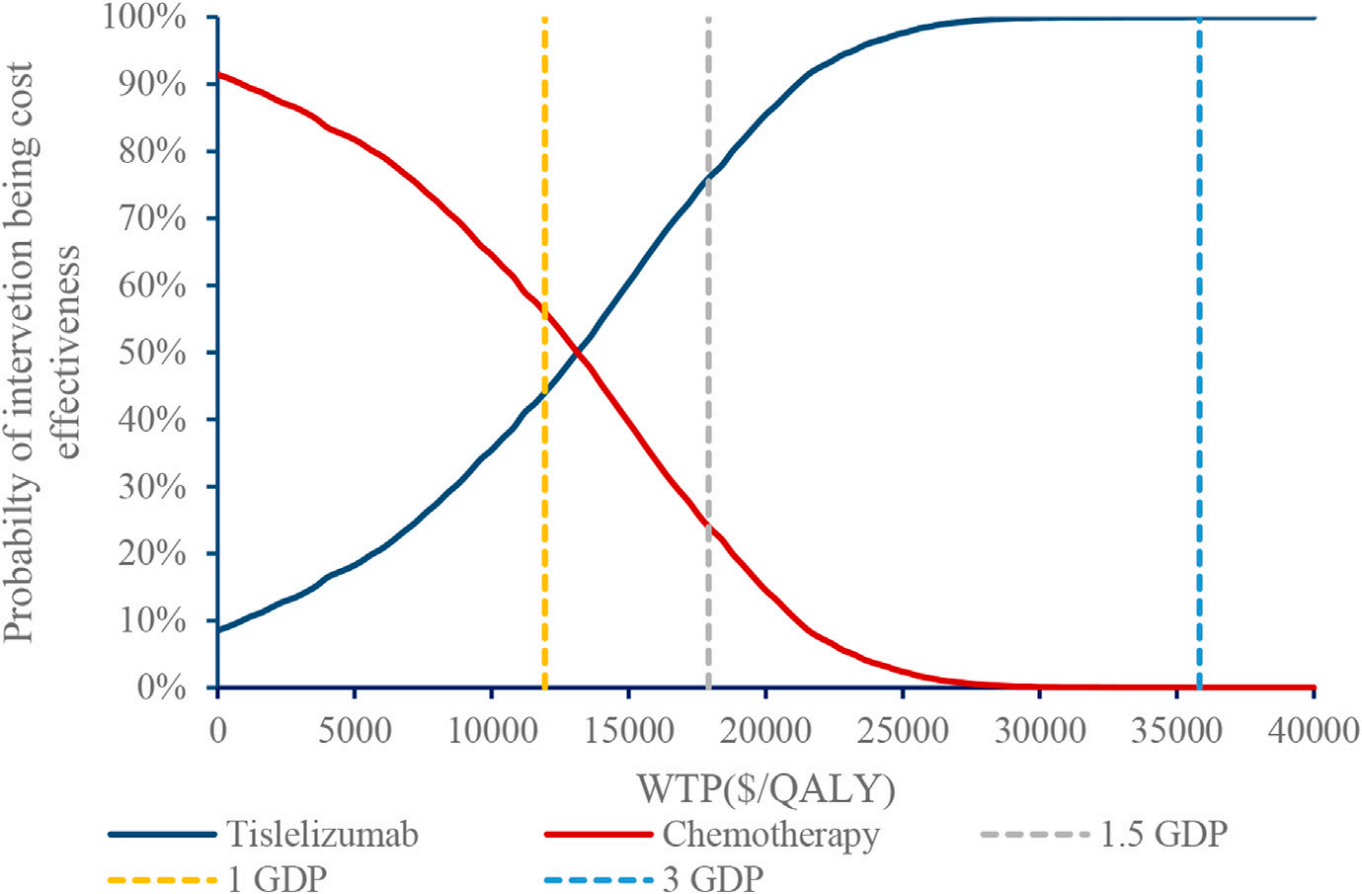
Cost-effectiveness acceptability curve.

Figure 3 shows the incremental ICER scatterplot of 5000 Monte Carlo iterations. Most ICERs fell in the northeast quadrant of the plane, indicating that tislelizumab resulted in a better effect at a higher cost than chemotherapy. The CEAC further illustrated that the tislelizumab regimen had 44%, 76%, and 100% probabilities of being economical at WTP thresholds of $11943/QALY (1 times GDP), $17915/QALY (1.5 times the GDP) and $35830/QALY (3 times the GDP), respectively (Figure 4).

## Discussion

This study is the first cost-effectiveness analysis of tislelizumab for ESCC from the Chinese healthcare payers’ perspective. Due to the high cost of immune checkpoint inhibitors (ICIs), it is unclear whether the tislelizumab would be economical for advanced ESCC. Hence, it is crucial to evaluate the effect of tislelizumab in china. We obtained relevant OS and PFS data from a diligently published phase III clinical trial of tislelizumab. The base-case analysis indicated that compared with chemotherapy therapy, the ICER of tislelizumab for second-line treatment of advanced or metastatic ESCC in China was $11073.85/QALY. According to the results of DSA, the cost of irinotecan and tislelizumab were driving factors of the evaluation. In the PSA, the tislelizumab regimen had 76% probability of being economical at WTP thresholds of 1.5 times GDP per capita in China, ensuring the economic advantage of tislelizumab and the stability of the model.

The current economic evaluation studies on immunotherapy for the second-line treatment of advanced or metastatic ESCC mainly concentrated on three drugs: nivolumab, camrelizumab, and pembrolizumab. Economic evaluations of nivolumab and pembrolizumab compared with chemotherapy based on the ATTRACTION-3 and the KEYNOTE-181 trial showed that both nivolumab and pembrolizumab were not economical compared with chemotherapy in China (Zhang et al., 2020; Zhan et al., 2022). The main reason for this disparity is that nivolumab and pembrolizumab are not included in the National Medical Insurance catalogue. For camrelizumab, the economic evidence was inconsistent as its entrance to China’s National Medical Insurance catalogue vastly improved its probability of being cost-effective compared with chemotherapy for advanced or metastatic ESCC in China (Cai H. et al., 2021; Lin et al., 2021; Yang et al., 2021; Li et al., 2022).

The study is subject to several limitations. First, since the RATIONALE-302 study did not publish utility data, we retrieved utility data from the literature, which might cause a certain degree of bias due to the inconsistency of study participants. However, we conducted the one-way sensitivity analysis of utility values and found that the study ICERs are not sensitive to utility values. Second, the RATIONALE-302 trial did not report the dosing regimen for patients after progression, so we assume that patients on tislelizumab and chemotherapy have the same follow-up regimen and are on the best supportive care. The cost of the best supportive care was adopted from previous pharmacoeconomics research for Chinese patients with advanced ESCC in 2021 (Cai D.et al., 2021). Third, due to the lack of individual data from the RATIONALE-302 trial, we did not perform a subgroup analysis to demonstrate the heterogeneity of patients’ characteristics. Finally, we did not compare other immunotherapy strategies for the second-line treatment model for ESCC, such as nivolumab (Kato et al., 2019), camrelizumab (Huang et al., 2020), and pembrolizumab (Kojima et al., 2020) as it requires further analysis using network meta-analysis.

## Conclusion

Based on the base case and sensitivity analyses, tislelizumab therapy was highly cost-effective compared with chemotherapy therapy, and it could be a promising strategy for treating ESCC patients under the Chinese setting. The results of this study could be a valuable reference for decision-makers and clinical practitioners to expand the use of tislelizumab as a second-line treatment for advanced or metastatic ESCC.

## Data Availability

The raw data supporting the conclusion of this article will be made available by the authors, without undue reservation.
