# Supplementary material for: Economic evaluation of tislelizumab versus chemotherapy as second-line treatment for advanced or metastatic esophageal squamous cell carcinoma in China
Source: Front Pharmacol. 2022 Nov 16;13:961347. doi: 10.3389/fphar.2022.961347 (PMC9708733; doi:10.3389/fphar.2022.961347)
Supplement: Supplementary file 1 [file Table1.DOCX]

**Title:** Cost-Effectiveness Analysis of Sintilimab + Chemotherapy versus Camrelizumab + Chemotherapy for the Treatment of First-Line Locally Advanced or Metastatic Nonsquamous NSCLC in China

**Authors:** Fenghao Shi^1,2^, Zixuan He^1,2^, Hang Su^1,2^, Lin Wang^3^, Sheng Han^1,2^

1 International Research Center for Medicinal Administration, Peking University, Beijing, China;

2 School of Pharmaceutical Sciences, Peking University, Beijing, China;

3 School of International Pharmaceutical Business, China Pharmaceutical University, Nanjing, China

Supplemental Table 1 Baseline Characteristics^[1]^.

| Population Characteristic | Tislelizumab (n=256) | Chemotherapy (n=256) |
| --- | --- | --- |
| Age, years, median (range) | 62.0 (40-86) | 63.0 (35-81) |
| <65, No. (%) | 157 (61.3) | 161 (62.9) |
| ≥65, No. (%) | 99 (38.7) | 95 (37.1) |
| Sex, No. (%) |  |  |
| Male | 217 (84.8) | 215 (84.0) |
| Female | 39 (15.2) | 41 (16.0) |
| Race, No. (%) |  |  |
| Asian | 201 (78.5) | 207 (80.9) |
| White or Caucasian | 53 (20.7) | 44 (17.2) |
| Black or African American | 0 (0.0) | 2 (0.8) |
| Other | 0 (0.0) | 1 (0.4) |
| Not reported/unknown | 2 (0.8) | 2 (0.8) |
| Geographic region, No. (%) |  |  |
| Asia | 201 (78.5) | 203 (79.3) |
| Europe/North America | 55 (21.5) | 53 (20.7) |
| ECOG PS, No. (%) |  |  |
| 0 | 66 (25.8) | 60 (23.4) |
| 1 | 190 (74.2) | 196 (76.6) |
| PD-L1 expression, No. (%) |  |  |
| TAP ≥ 10% | 89 (34.8) | 68 (26.6) |
| TAP <10% | 116 (45.3) | 140 (54.7) |
| Unknown | 51 (19.9) | 48 (18.8) |
| Smoking status, No. (%) |  |  |
| Never | 68 (26.6) | 63 (24.6) |
| Former/current | 188 (73.4) | 192 (75.0) |
| Missing | 0 (0.0) | 1 (0.4) |
| Previous therapies, No. (%) |  |  |
| Surgery | 94 (36.7) | 99 (38.7) |
| Radiotherapy | 169 (66.0) | 163 (63.7) |
| Platinum-based chemotherapy | 249 (97.3) | 252 (98.4) |
| Disease stage at study entry, No. (%) |  |  |
| Locally advanced | 5 (2.0) | 20 (7.8) |
| Metastatic | 251 (98.0) | 236 (92.2) |

Supplemental Table 2. AIC and BIC of curve fitting for OS and PFS

|  | OS in tislelizumab | | PFS in tislelizumab | | OS in chemotherapy | | PFS in chemotherapy | |
| --- | --- | --- | --- | --- | --- | --- | --- | --- |
|  | AIC | BIC | AIC | BIC | AIC | BIC | AIC | BIC |
| Exponential | 1391.895 | 1395.441 | 1115.587 | 1119.133 | 1384.168 | 1387.714 | 817.787 | 821.332 |
| Weibull | 1390.906 | 1397.996 | 1114.339 | 1121.43 | 1371.893 | 1378.983 | 805.792 | 812.882 |
| Gamma | 1388.828 | 1395.918 | 1117.578 | 1124.668 | 1365.464 | 1372.555 | 793.316 | 800.407 |
| Log logistic | **1381.079** | **1388.17** | 1037.139 | 1044.229 | **1353.719** | **1360.809** | 761.750 | 768.841 |
| Log normal | 1382.894 | 1389.985 | **1036.125** | **1043.215** | 1356.344 | 1363.435 | 756.454 | 763.545 |
| Gompertz | 1393.887 | 1400.977 | 1081.53 | 1088.62 | 1384.955 | 1392.046 | 819.758 | 826.848 |
| Generalized gamma | 1383.175 | 1393.81 | **985.8029** | **996.4384** | 1357.005 | 1367.64 | **749.433** | **760.069** |

OS: Overall survival, PFS: Progression free survival, AIC: Akaike Information Criteria, BIC: Bayesian Information Criteria

Supplemental Table 3. Adverse Events Rates and Costs Inputs

|  | Event rate %  (tislelizumab) | Event rate %  (chemotherapy) | Cost | Disutility | Source |
| --- | --- | --- | --- | --- | --- |
| Anemia | 11 | 34.6 | 138.03 | 0.1637 | ^[2, 3]^ |
| Diarrhea | 5.5 | 27.5 | 3.28 | 0.0499 | ^[2, 3]^ |
| Nausea | 2.7 | 27.5 | 27.15 | 0.0464 | ^[2, 4]^ |
| WBC count decreased | 2 | 40.8 | 114.41 | 0.0000 | ^[2, 4]^ |
| Vomiting | 1.6 | 17.9 | 98.33 | 0.0607 | ^[2, 5]^ |
| Neutrophil count decreased | 1.2 | 39.2 | 114.41 | 0.0000 | ^[2, 5]^ |
| Neutropenia | 0.8 | 12.9 | 114.41 | 0.0916 | ^[2 3]^ |

Supplemental Fig S1. Long-term overall survival for tislelizumab


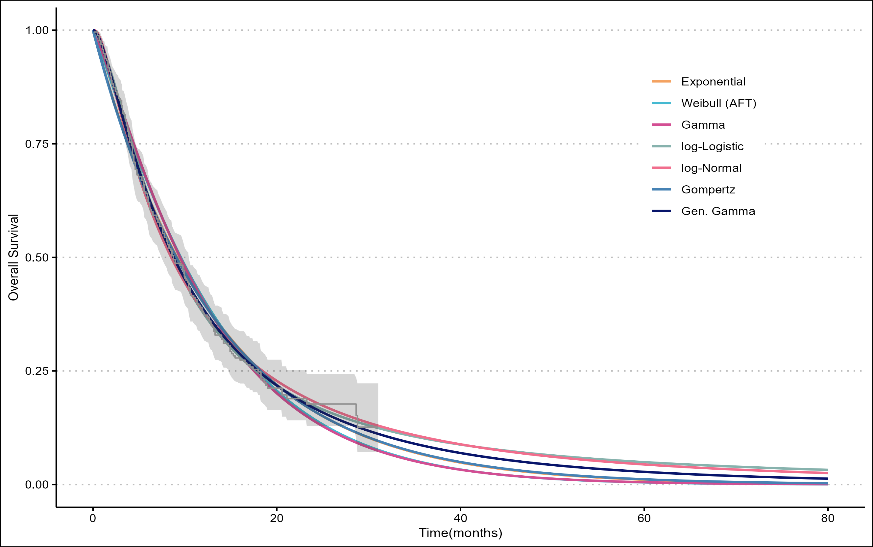


Supplemental Fig S2. Long-term progression-free survival for tislelizumab


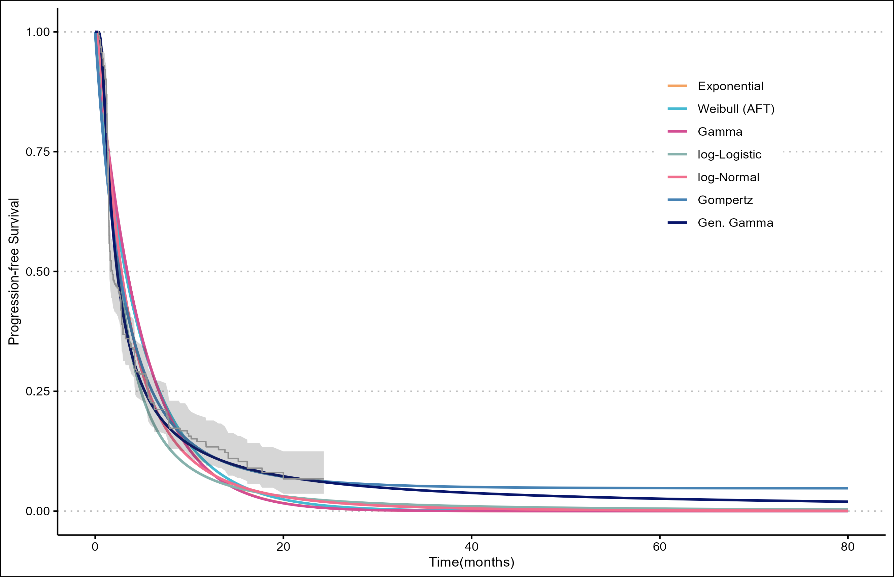


Supplemental Fig S3. Long-term overall survival for chemotherapy


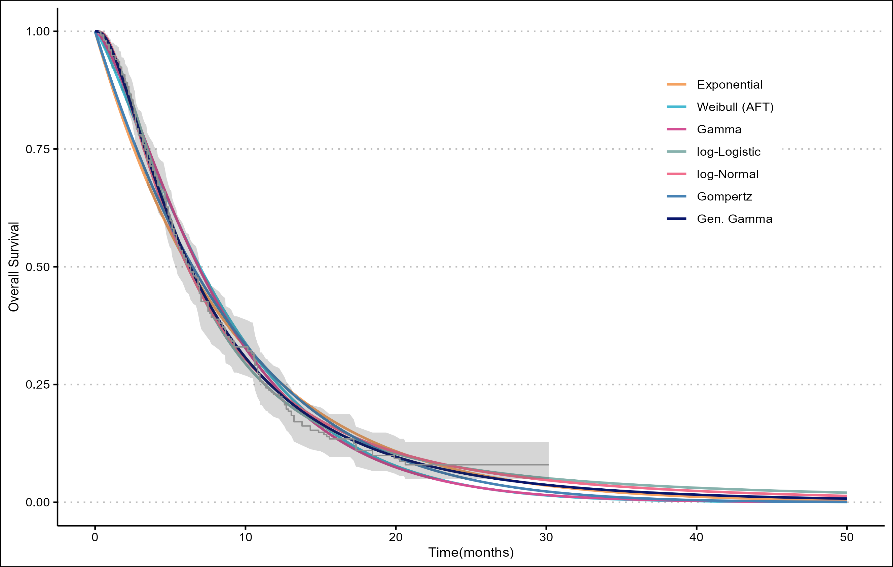


Supplemental Fig S4. Long-term progression-free survival for chemotherapy


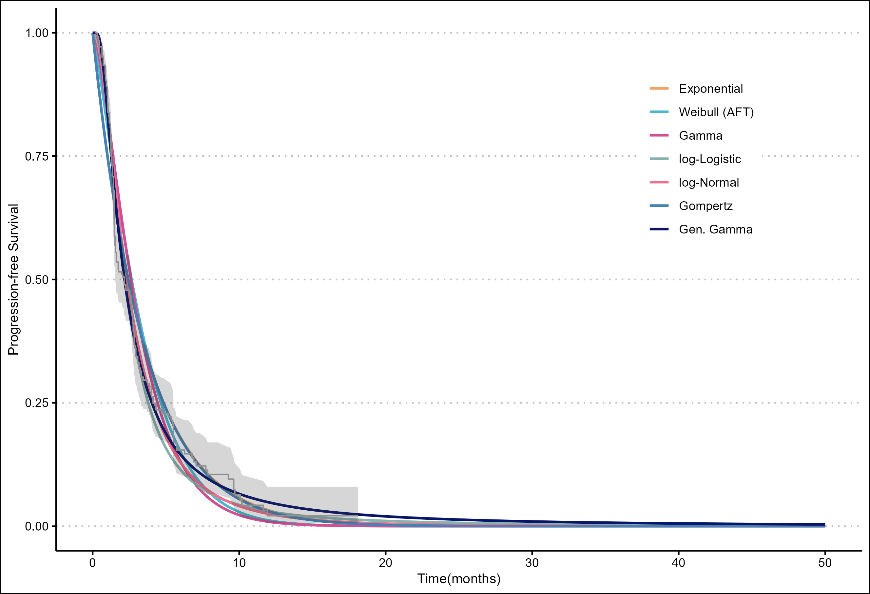


**References**

1 Shen, L., Kato, K., Kim, S., Ajani, J. A., Zhao, K., He, Z., Yu, X., Shu, Y., Luo, Q., and Wang, J. (2022). Tislelizumab Versus Chemotherapy as Second-Line Treatment for Advanced or Metastatic Esophageal Squamous Cell Carcinoma (RATIONALE-302): A Randomized Phase III Study. J. Clin. Oncol., JCO. 21.01926

2 Zhou T, Cao Y, Wang X, et al. Economic Evaluation of Sintilimab Plus Bevacizumab Versus Sorafenib as a First-line Treatment for Unresectable Hepatocellular Carcinoma[J]. Advances in Therapy, 2022,39(5):2165-2177.

3 Saiyed M, Byrnes J, Srivastava T, et al. Cost-effectiveness of lenvatinib compared with sorafenib for the first-line treatment of advanced hepatocellular carcinoma in Australia[J]. Clinical Drug Investigation, 2020,40(12):1167-1176.

4 Su D, Wu B, Shi L. Cost-effectiveness of atezolizumab plus bevacizumab vs sorafenib as first-line treatment of unresectable hepatocellular carcinoma[J]. JAMA network open, 2021,4(2):e210037.

5 Kim J J, McFarlane T, Tully S, et al. Lenvatinib Versus Sorafenib as First‐Line Treatment of Unresectable Hepatocellular Carcinoma: A Cost–Utility Analysis[J]. The oncologist, 2020,25(3):e512-e519.
